# A method for accurate spatial registration of PET images and histopathology slices

**DOI:** 10.1186/s13550-015-0138-7

**Published:** 2015-11-14

**Authors:** Tanuj Puri, Anastasia Chalkidou, Rhonda Henley-Smith, Arunabha Roy, Paul R. Barber, Teresa Guerrero-Urbano, Richard Oakley, Ricard Simo, Jean-Pierre Jeannon, Mark McGurk, Edward W. Odell, Michael J. O’Doherty, Paul K. Marsden

**Affiliations:** Division of Imaging Sciences and Biomedical Engineering, King’s College London, London, UK; Present address: Department of Oncology, University of Oxford, Oxford, UK; Oral Pathology Department, King’s College London, London, UK; Department of Oncology, Cancer Research UK and Medical Research Council Oxford Institute for Radiation Oncology, University of Oxford, Oxford, UK; Institute for Mathematical and Molecular Biomedicine, King’s College London, London, UK; Department of Clinical Oncology, Guy’s & St Thomas’ NHS Foundation Trust, London, UK; Department of Head & Neck Surgery, Guy’s & St Thomas’ NHS Foundation Trust, London, UK

**Keywords:** Histopathology, PET, Registration, Methodology, Oncology

## Abstract

**Background:**

Accurate alignment between histopathology slices and positron emission tomography (PET) images is important for radiopharmaceutical validation studies. Limited data is available on the registration accuracy that can be achieved between PET and histopathology slices acquired under routine pathology conditions where slices may be non-parallel, non-contiguously cut and of standard block size. The purpose of this study was to demonstrate a method for aligning PET images and histopathology slices acquired from patients with laryngeal cancer and to assess the registration accuracy obtained under these conditions.

**Methods:**

Six subjects with laryngeal cancer underwent a ^64^Cu-copper-II-diacetyl-bis(N4-methylthiosemicarbazone) (^64^Cu-ATSM) PET computed tomography (CT) scan prior to total laryngectomy. Sea urchin spines were inserted into the pathology specimen to act as fiducial markers. The specimen was fixed in formalin, as per standard histopathology operating procedures, and was then CT scanned and cut into millimetre-thick tissue slices. A subset of the tissue slices that included both tumour and fiducial markers was taken and embedded in paraffin blocks. Subsequently, microtome sectioning and haematoxylin and eosin staining were performed to produce 5-μm-thick tissue sections for microscopic digitisation. A series of rigid registration procedures was performed between the different imaging modalities (PET; in vivo CT—i.e. the CT component of the PET-CT; ex vivo CT; histology slices) with the ex vivo CT serving as the reference image. In vivo and ex vivo CTs were registered using landmark-based registration. Histopathology and ex vivo CT images were aligned using the sea urchin spines with additional anatomical landmarks where available. Registration errors were estimated using a leave-one-out strategy for in vivo to ex vivo CT and were estimated from the RMS landmark accuracy for histopathology to ex vivo CT.

**Results:**

The mean ± SD accuracy for registration of the in vivo to ex vivo CT images was 2.66 ± 0.66 mm, and the accuracy for registration of histopathology to ex vivo CT was 0.86 ± 0.41 mm. Estimating the PET to in vivo CT registration accuracy to equal the PET-CT alignment accuracy of 1 mm resulted in an overall average registration error between PET and histopathology slices of 3.0 ± 0.7 mm.

**Conclusions:**

We have developed a registration method to align PET images and histopathology slices with an accuracy comparable to the spatial resolution of the PET images.

## Background

Positron emission tomography (PET) is a molecular imaging technique used for the visual and quantitative assessment of a tissue of interest via administration of a radiopharmaceutical tracer. The validation of a new tracer can be achieved by comparing the tracer uptake seen in PET images with the gold standard of histopathology imaging. Once validated, the new tracer can then be used to provide information about diagnosis, prognosis and response to treatment. Due to the heterogenous nature of tumours and the consequent heterogenous distribution of tracer uptake [1], the validation of PET against histopathology images can only be considered meaningful if spatially corresponding regions are compared.

The process of spatially aligning multi-modal image data sets is challenging and is usually achieved using anatomical landmarks, features and/or pixel intensity values to establish correspondences between images acquired with different modalities. The registration between in vivo human tomographic images and three-dimensional (3D) histopathology data in the brain has been shown to obtain satisfactory results with submillimetre accuracy [2, 3]. However, the process of obtaining 3D reconstructions of the whole histopathology specimen is laborious, expensive and not feasible in most routine pathology settings. The post processing for such an approach includes (a) registration of individual histopathology slices to block-face images (photographic images of the specimen taken before and after cutting) to correct for the shrinkage incurred during histopathology procedures [4], (b) registration of shrinkage-corrected histopathology slices to each other to reconstruct a 3D volume [5, 6] and (c) registration of the histopathology volume to the in vivo imaging data (i.e. in vivo PET/computed tomography (CT)/MR) using intensity-based or other algorithms directly [2]. In some cases, the in vivo imaging and histology volume are registered using volumetric block-face images as the registration reference [3] due to the latter’s rich spatial information content. However, the 3D reconstruction of block-face images can be distorted by the so-called ‘banana effect’ whereby a 3D curved object cannot be reconstructed from cross-sections without any additional information [7]. It is possible to minimise related registration errors by using an additional intermediate tomographic scan, for example, CT or MRI, of the unsliced specimen (i.e. an ex vivo scan) with inserted fiducial markers that can be visualised on both ex vivo and block-face images to drive the reconstruction of the 3D block-face volume [4]. Shojaii R. et al. [8] used a catheter filled with cuttlefish ink and flour as a fiducial marker that was visible in multi-modal images. A number of other markers have also been proposed, for example, cotton thread infused with solution of gadopentetate dimeglumine and blue tissue marking dye [9], plastic sheaths of catheter needles [10], cotton thread infused with a solution of Magnevist and blue tissue marking dye [11] and bronze and stainless-steel paints [12]. However, most of these markers/studies are MRI specific.

In addition, the use of systematic sectioning of the specimen, to obtain millimetre-thick tissue slices, simplifies the cutting of parallel and consecutive slices [9], as opposed to using a freehand technique. Mega et al. [13] registered stained whole-brain sections to pre-mortem PET images in Alzheimer’s disease, however, no registration errors were reported. Edwards et al. [14] presented a system for PET-to-histology registration for image-guided surgery but did not report any registration errors. A few other studies involved the registration of histology with autoradiographs in preclinical settings [3, 15–18] and/or histology with MRI [2, 9, 12, 19–21].

Parallel sectioning of the specimen provides good thickness estimates of the sectioned slices, contiguous sectioning aids in minimising the errors in reconstructing the 3D histopathology volume and whole-tissue histopathology slices provide confidence in matching boundaries with block-face/in vivo/ex vivo data. However, registration of non-parallel, non-contiguously cut and non-whole-sized histopathology slices to, for example, tomographic PET slices is more difficult, particularly in regard to identifying corresponding slices in the axial (i.e. perpendicular to the cutting plane) direction. Because the PET and CT data are inherently 3D and the histology slices are inherently two dimensional (2D), an important aspect of the PET and ex vivo CT to histology registration is the identification of corresponding *z*-axis levels. The difficulty in identifying the *z*-axis accurately emerges from the lack of a 3D reconstruction of the specimen volume after slicing. Although the specimen is cut into consecutive slices, standard pathology procedures do not involve the use of a system that ensures all slices are cut to exactly the same thickness. As a consequence, a histology slice might not be cut perpendicular to the *z*-axis of the specimen and different areas of the micron-thick histology data could belong to different millimetre-thick slices.

Therefore, the aim of this work was to develop a registration methodology for an accurate spatial alignment between PET/CT images of the head and neck region in humans and histopathology slices obtained in routine pathology settings where slices may be not perfectly parallel, non-contiguously cut and non-whole-slice sized.

## Methods

### Subjects

Eligible patients for this prospective study were identified at the head and neck oncology multidisciplinary meetings and recruited from the head and neck oncology clinic at Guy’s and St Thomas’ Hospital. Patients were eligible to take part if they were scheduled to undergo total laryngectomy with or without lymph node dissection. Staging of the tumours was performed according to the TNM staging system. From March 2011 to December 2012, eight consecutive patients were prospectively enrolled into the study. They were all patients with advanced-stage HNSCC laryngeal cancer, and six patients were included in the final analysis. Two patients were excluded as they did not undergo a ^64^Cu-ATSM PET/CT scan—one due to changes in his clinical management and one due to declining to complete the scan after recruitment. The specific patient and tumour characteristics for both cohorts are summarised in Table 1.

**Table 1:** Patient and tumour characteristics

Patient ID	Age	TNM	Surgery
P1	66	T4N2cM0	Total laryngectomy and bilateral neck dissection
P2	62	T3N2cM0	Total laryngectomy and bilateral neck dissection
P3	65	T4aN3M0	Total laryngectomy and bilateral neck dissection
P4	70	T4aN2bM0	Total laryngectomy and bilateral neck dissection
P5	73	T4N2bM0	Total pharyngolaryngectomy and bilateral neck dissection
P6	75	T4N0M0	Total laryngectomy and bilateral selective neck dissection

All lesions were histopathologically confirmed based on biopsy results. In total, eight lesions (six primary tumours and two lymph nodes) were identified as disease positive. The study was approved by the London Bridge Research Ethics Committee (reference 07/Q0704/8, 1/2011). All patients signed an informed consent form agreeing to participate in the study and to publication of results. A copy of the study protocol can be found by following the link [22].

### Study design

**Fig. 1:**
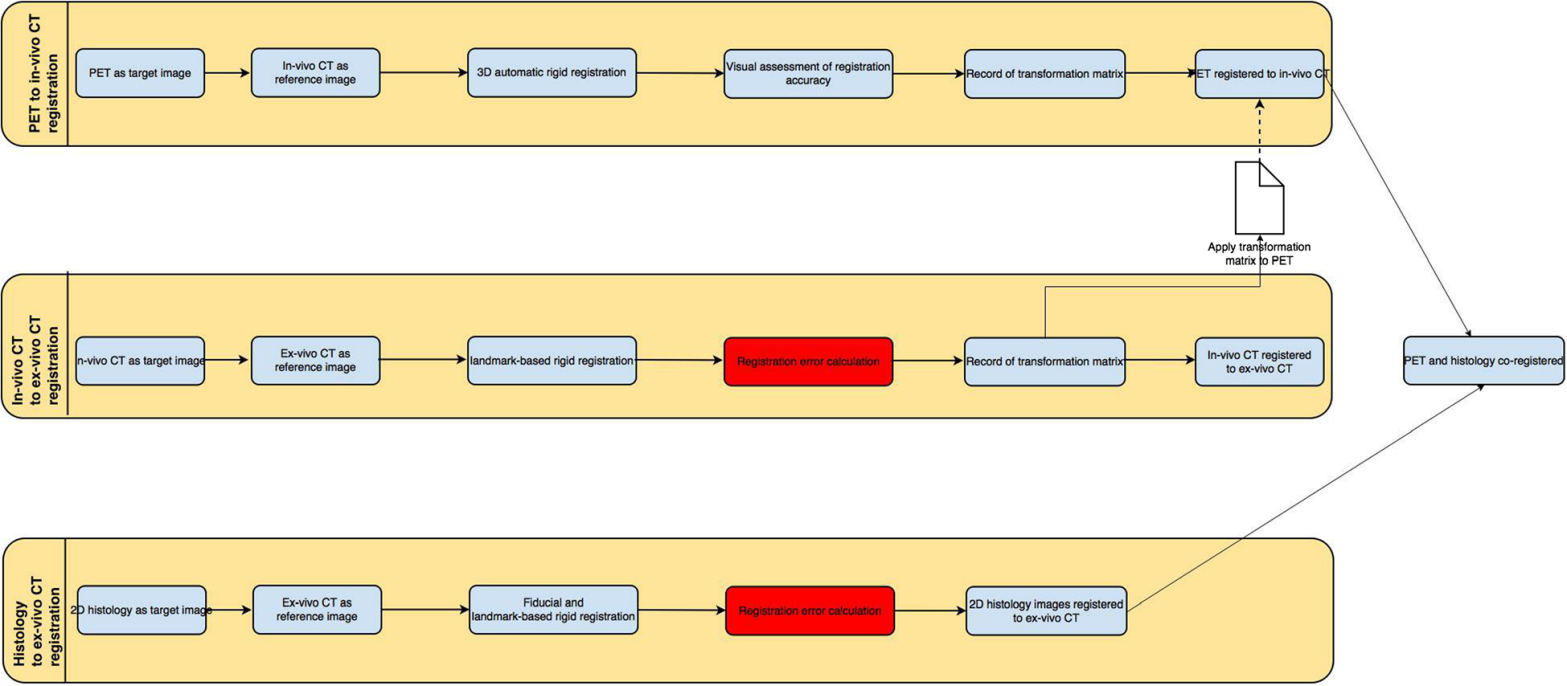
Registration framework. The various steps in the registration methodology framework applied in this study. *PET* positron emission tomography, *CT* computed tomography, *H&E* haematoxylin and eosin

The registration framework followed in this study is shown in Fig. 1, and imaging parameters are outlined in Table 2. Each of the steps is explained in detail as follows:

**Table 2:** Summary of imaging parameters

Imaging modality	In-plane reconstruction FOV (mm)	Pixel size in *XYZ* (mm)	3D or 2D
PET	500	3.91 × 3.91 × 3.27	3D
CT in vivo	700	1.37 × 1.37 × 3.27	3D
CT ex vivo	Specimen fit (approximately few cm)	0.71 × 0.71 × 1.00	3D
Block face	Specimen fit (approximately few cm)	0.25 × 0.25	2D
Histology	Specimen fit (approximately 2 cm)	0.00134 × 0.00134	2D

*FOV* field of view, *XYZ x*-axis, *y*-axis and *z*-axis, respectively

#### PET-CT scanning

The ^64^Cu-copper-II-diacetyl-bis(N4-methylthiosemicarbazone) (^64^Cu-ATSM) PET computed tomography (CT) scans were acquired a week before the surgery on a PET/CT GE VCT scanner (General Electric Medical Systems, Waukesha, WI, USA) with 500-mm axial field of view (FOV) and spatial resolution of 6-mm full-width-at-half-maximum (FWHM) [23]. Subjects were scanned in fasting state and positioned supine, arms down wi th knee support such that the tumour site was in the centre of the FOV. Administered was 600 MBq of ^64^Cu-ATSM, a putative marker of tissue hypoxia, followed by a low-dose CT scan and a 10-min PET scan 70 min post-injection. The patient’s head was fixed firmly but comfortably to minimise movement during the PET-CT scan using the standard scanner headrest. Five out of six PET data sets were reconstructed using the three-dimensional (3D) re-projection algorithm and one data set using the proprietary GE 3D-‘Viewpoint’ algorithm. All PET images were corrected for radioactive decay and attenuation corrected using CT.

#### Fiducial markers

**Fig. 2:**
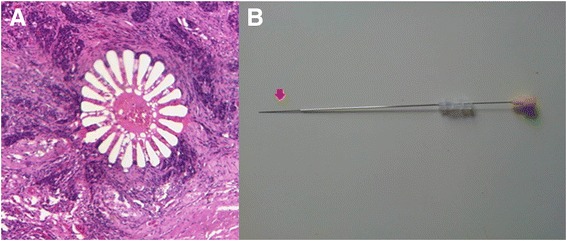
Sea urchin spine fiducial markers. **a** Example of a 5-μm-thick tissue section showing a sea urchin spine in the transverse plane in a haematoxylin and eosin (H&E)-stained histopathology slice at a spatial resolution of 1 μm/pixel. **b** 20-gauge spinal needle loaded with the sea urchin spine fiducial marker. The tip of the spine can be seen protruding from the needle (*red arrow*)

**Fig. 3:**
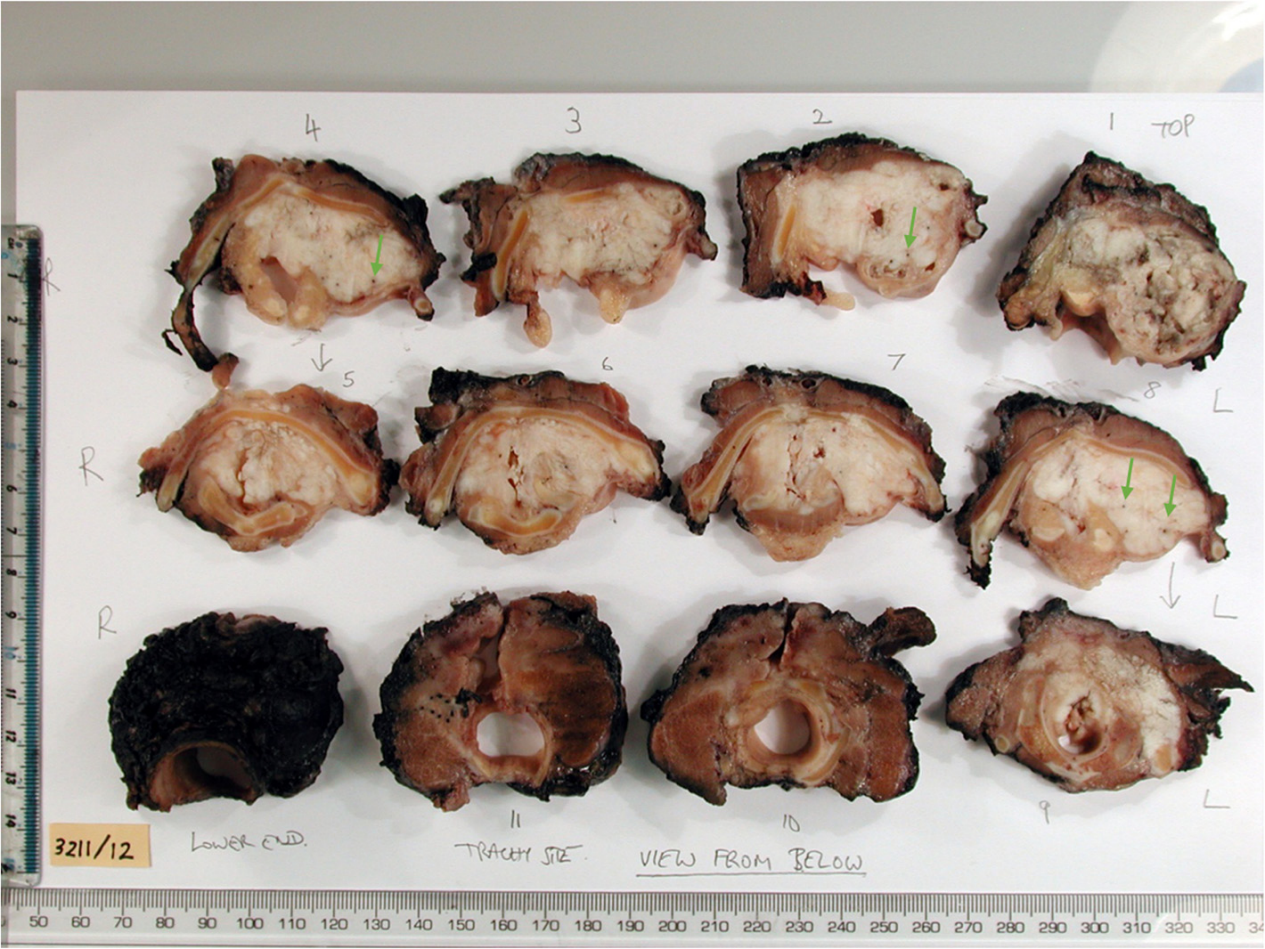
Laryngeal pathology specimen. Example of a laryngeal pathology specimen from a patient with advanced laryngeal cancer that was included in the study. The exact anatomical orientation (left to right and top to bottom) and the consecutive cutting of the samples are displayed, as recorded at the time of the slicing by the pathology lab staff

The logistics and feasibility of using spines of black sea urchin as fiducial markers in human specimens were first tested ex vivo. Markers were tested for length and diameter, insertion into a specimen, ease of cutting with minimal tissue tearing and visibility in histopathological sections. The spines consist of calcium carbonate (CaCO_3_) and magnesium carbonate (MgCO_3_), and they are therefore visible during CT scanning [24]. Spines of length 20 to 30 mm and diameter 0.5 mm (approximately) (Fig. 2a) were inserted into the specimen using a 20-gauge and 3.5-in. Quincke spinal needle (Fig. 2b) with stylet and cannula hubs (Becton, Dickinson and Company, Rutherford, New Jersey, USA). The spines were pushed into the fresh specimen using the stylet, and the cannula was retracted at the same time such that the spine remained in the desired tumour location where the needle was initially inserted. Spines were inserted at angles to each other (typically ~30°) in order to facilitate slice identification (see below). Figure 2a shows the appearance of a spine on a haematoxylin and eosin (H&E)-stained histopathology slice at a spatial resolution of 1 μm/pixel. The spines were also visible on ex vivo CT and block-face images (Fig. 3). For technical reasons, only four out of six specimens were inserted with fiducial markers and so were able to be registered with PET.

#### Specimen fixation and ex vivo CT scanning

The specimen was fixed in 10 % formalin for 24 h after marker insertion. Block-face images of the whole specimen were obtained before and after fixation using an optical/digital camera (Nikon Coolpix 995, Nikon, Japan). The block-face images served as visual reference for the registration process. The specimen was then scanned ex vivo on the CT component of a SPECT-CT scanner (Philips Precedence 16, Eindhoven, Netherlands) with careful positioning such that the orientation differences between in vivo and ex vivo CT scans of the larynx were minimised.

#### Specimen slicing and block-face imaging

The larynx specimen was sliced into ~5-mm thick slices using a bandsaw (Exakt saw, Norderstedt, Germany) with a blade thickness of 300 μm. The average ± SD thickness of all the slices (excluding first and last slice) from five specimens was 5.28 ± 2.42 mm. Tissue slices from every specimen were laid out on a flat glass table lit with luminous white light (from above and underneath) to obtain a high-contrast image of both sides. The camera position was fixed above the table such that all tissue slices of a specimen were captured in a single shot.

#### Histopathology procedure and microscopic digitisation

**Fig. 4:**
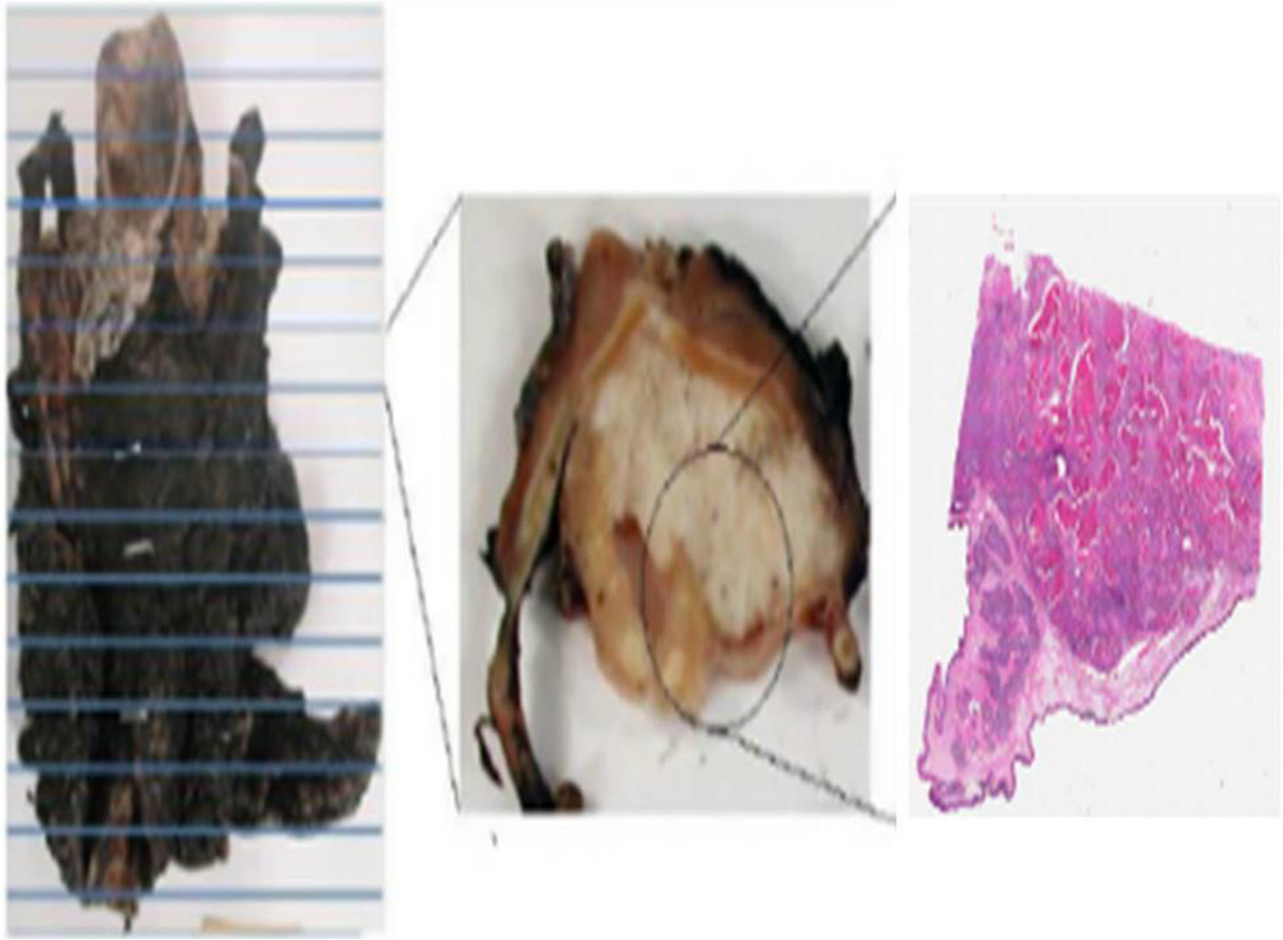
Slicing of excised specimen. A schematic diagram showing (*left*) the excised specimen, (*centre*) a ~5-mm-thick tissue slice and (*right*) a 5-μm-thick tissue slice that has been stained. A relevant section is taken from the 5-mm slice which is then embedded in paraffin prior to microtome cutting to obtain the 5-μm-thick tissue slices

One or more subsections of every 5-mm-thick tissue slice that included tumour and fiducial markers were cut and embedded in paraffin blocks followed by microtome cutting. Microtome cutting results in the production of 5-μm-thick tissue slices that are subsequently attached to a glass slide of size 30 × 21 mm and stained with H&E. Finally, they were digitised using a light microscope (Lister ‘Open’ Microscope, CRUK/MRC Oxford Institute for Radiation Oncology, Oxford, UK). The microscope is based on a TE200 body with a ×4 (NA 0.16) air objective lens (Nikon, UK) and comprises a 3-CCD colour camera (KY-F75U, JVC UK), motorised stage (Scan IM 120 × 100, Marzhauser, Germany) and in-house written software [25]). The in-plane FOV of the microscope was set to acquire the whole slide in multiple stages at a spatial resolution of 1 μm/pixel, and individual images were stitched together with 5 % overlapping such that the edges matched and became invisible. A total of twenty-three 5-μm-thick histopathology slices were obtained from six laryngeal specimens (eighteen 5-μm-thick slices belonged to the four specimens with sea urchin spine fiducials inserted). Of the twenty-three slices, two slices had 4 fiducial markers each, six slices had 3 markers each, five slices had 2 markers each, one slice had 1-marker and nine slices had no marker. Figure 4 shows the thickness differences between the imaging and 3D histopathology data and the final 5-μm-thick tissue slices.

### Image registration procedures

A series of four rigid image registration steps, described in detail below, was performed to align PET images and histopathology slices. The ex vivo CT was used as the reference image such that PET, in vivo CT, ex vivo CT and histopathology were all in this same space after alignment. All registrations were performed using PMOD software (PMOD Technologies Ltd., Zurich, Switzerland).

#### Step 1—registration of PET to in vivo CT

PET and in vivo CT images are both acquired as part of the combined PET-CT scan. This step was performed using 3D rigid registration with a normalised mutual information objective and visually checked by a clinician (AC—6 years clinical oncology and 4 years PET/CT experience).

#### Step 2—registration of in vivo CT to ex vivo CT

**Fig. 5:**
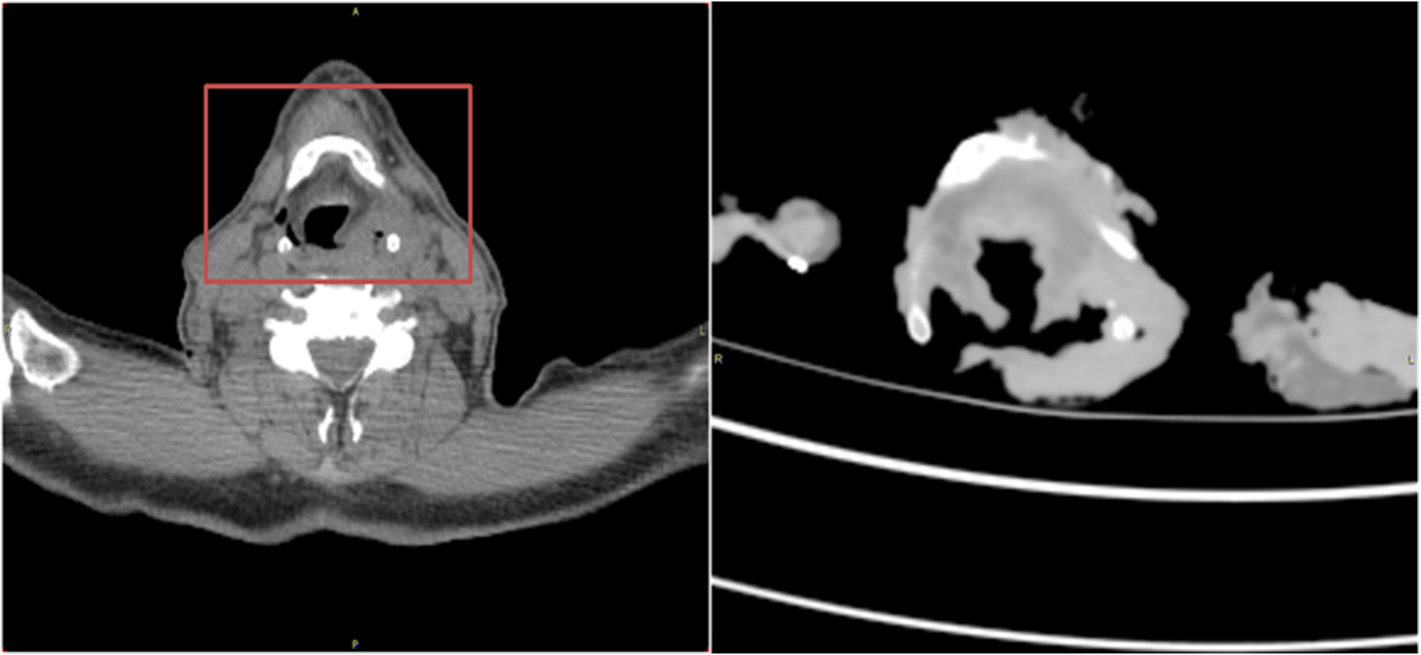
In vivo CT (*left*) and the corresponding ex vivo CT (*right*) of the pathology specimen. The ex vivo CT corresponds to the area outlined by the *red box*. Large deformations occurring due to the surgical excision can be seen

Thirteen anatomical landmarks were systematically identified on the thyroid and cricoid cartilage. These included the superior horns of the thyroid cartilage, the middle and posterior edges of the thyroid and cricoid cartilage, the superior thyroid notch and hyperdense areas where the cartilage was calcified and therefore easily identifiable. The landmarks were identified by a clinician (AC). Alignment was performed using a rigid point-based 3D registration procedure, and a transformation matrix was obtained for use in step 3. Figure 5 shows an example of an in vivo CT and the corresponding ex vivo CT of the pathology specimen.

#### Step 3—transformation of PET to ex vivo CT

The transformation matrix obtained in step 2 was applied to the PET image (previously registered to in vivo CT) such that the new transformed PET was in spatial alignment with the ex vivo CT.

#### Step 4—registration of histology to ex vivo CT

**Fig. 6:**
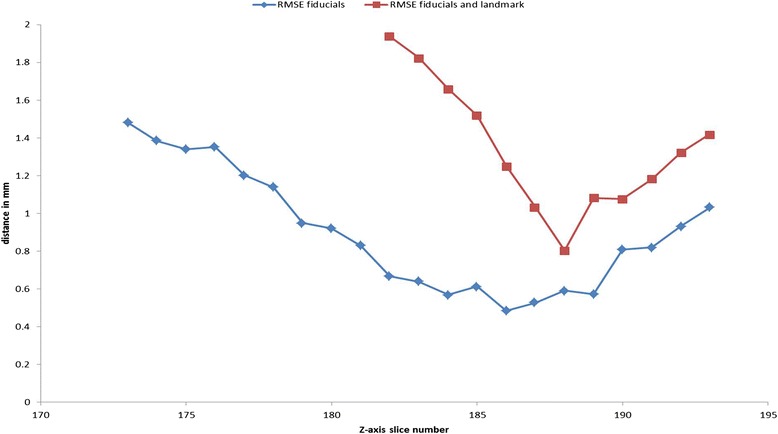
Identification of corresponding CT planes and histology slices. Example showing how corresponding histology slices and (ex vivo) CT planes are identified. The RMS distance is calculated using either four spine fiducial markers (*blue line*) or four spine fiducial markers and one anatomical landmark (*red line*). Although there is approximate agreement between the two methods, the use of landmarks leads to more precise identification as the landmarks are often confined to one or two consecutive axial slices. Although the measurements using the fiducial markers only (*blue line*) leads to smaller RMSE, both methods lead to a very similar position of the global minimum. The CT slice that showed corresponding to the minimum RMSE was chosen as that corresponding with the given histopathology slice. *RMSE* root mean square error

As the fiducial markers are long spines, they can be seen in many planes of both the histology slices and the ex vivo CT images. A histology slice was selected, and the in-plane root mean square (RMS) distance between fiducials, and any additional visible anatomical landmarks (minimum of three in total), in the histology slice and a CT slice was calculated and minimised using a 2D rigid registration. The residual RMS inter-marker distance was then plotted as a function of the CT slice number whilst the histology slice was kept constant. As the spine fiducials are aligned non-parallel to each other, the RMS distance increases rapidly for non-corresponding slices as shown in Fig. 6. The CT slice for which the RMS distance is a minimum is deemed to be the one corresponding to the current histology slice. In order to better identify the slice with minimum RMS inter-marker distance, a negative Gaussian function (of the form *a*−*b*.exp(−*x*^2^/*c*^2^) where *x* represents the CT slice number and *a*, *b* and *c* are fitted constants) was fitted to the inter-marker distance curves and the minimum point of the Gaussian was used as an estimate of the minimum inter-marker distance and the corresponding CT slice. This process was repeated for all other histology slices. In practice, the process described above was aided by careful documentation of the serial specimen cutting performed in the pathology department. The pathology department records precisely, as part of its diagnostic routine, the exact level where every tissue block is cut and collected from. This provides further detail of the *z*-level location with the help of images taken at the time of cutting the specimen into 5-mm slices (Fig. 3).

### Estimation of registration errors

#### Registration errors for PET and CT components of PET-CT

The error due to misalignment of the PET and CT components of the PET-CT scan was not measured and was assessed visually.

#### Registration errors for in vivo CT to ex vivo CT

The in vivo CT to ex vivo CT registration error was estimated using a ‘leave-one-out’ strategy as follows. The registration procedure described above was repeated 13 times leaving one pair of landmarks out each time to calculate 13 transformation matrices. Each left-out landmark on in vivo CT was then transformed using the corresponding transformation matrix. The distance between each transformed point and its corresponding point on the ex vivo CT was measured for each of the 13 cases. The error is then calculated as the average of all 13 values.

#### Registration errors for histology to ex vivo CT

Given the extended nature and small number of the fiducial markers, it was not possible to use a leave-one-out approach to estimate the histology to ex vivo CT registration error, and so, the error was approximated by the residual in-plane RMS inter-marker distance—note that this will not account for incorrectly matched histology and CT planes.

#### Total registration error

An estimate of the overall registration error between PET and histology was obtained by adding the three contributions (i.e. PET—in vivo CT; in vivo CT—ex vivo CT; ex vivo CT—histology) in quadrature.

## Results

### PET to in vivo CT co-registration

**Fig. 7:**
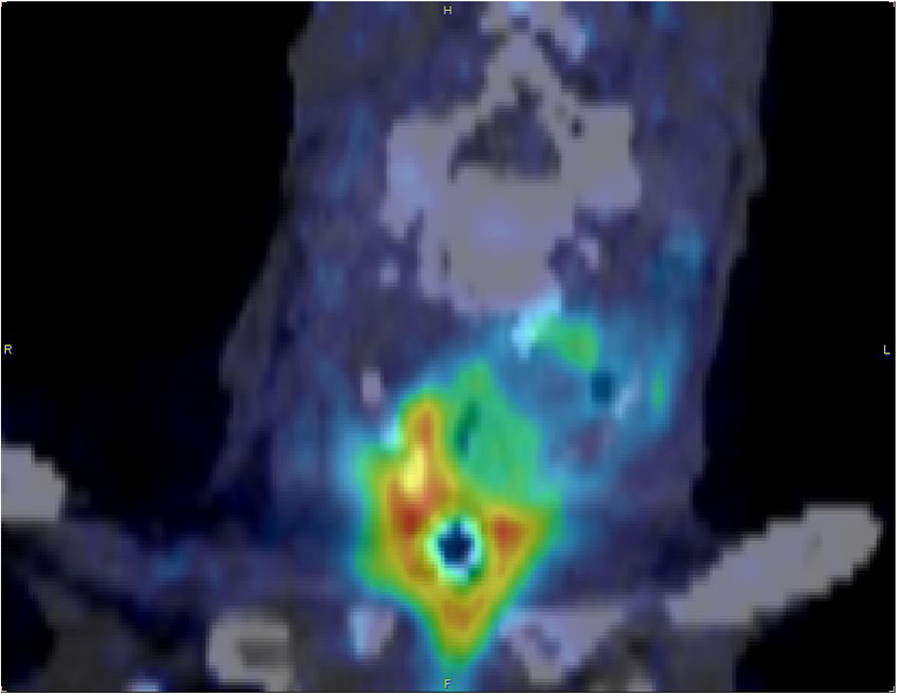
Fused PET and CT components of PET-CT scan. Coronal view of fused co-registered PET and ex vivo CT data for patient number 4. Very good alignment between the two data sets can be observed by using the tracheostomy as a reference point

All of the PET-CT data sets obtained from the scanner appeared well aligned and could not be corrected any further with registration (Fig. 7). This is consistent with the registration error being attributable to the scanner PET-CT alignment only which has an accuracy of ~1.0 mm [26].

### In vivo CT to ex vivo CT co-registration

Table 3 shows the results of in vivo CT to ex vivo CT registration error measurements. The average registration error between in vivo CT and ex vivo CT was 2.66 (SD: 0.66) mm.

**Table 3:** Registration accuracy

Subjects	CTin-CTex	CTex-histology	Total
P1-BH	NA	0.91	NA
P1-BJ	NA	1.86	NA
P1-BK	NA	1.52	NA
P2-B	NA	0.38	NA
P2-C	NA	0.92	NA
P3-BJ	3.55	1.08	3.71
P3-BH	3.55	0.58	3.60
P3-BK	3.55	0.11	3.55
P4-AA	2.83	0.8	2.94
P4-AB	2.83	0.85	2.95
P4-AC	2.83	0.97	2.99
P4-R	2.83	0.75	2.93
P5-M1	2.94	1.08	3.13
P5-M2	2.94	0.49	2.98
P5-S	2.94	1.14	3.15
P6-CC	1.84	1.09	2.14
P6-CD	1.84	0.89	2.04
P6-DB	1.84	0.50	1.91
P6-DC	1.84	0.62	1.94
P6-DD	1.84	0.83	2.02
Mean	2.66	0.86	2.80
SD	0.66	0.41	0.63

The table shows (columns from left to right) the subject number and corresponding histology samples, RMS registration error in millimetres resulting from landmark-based in vivo CT and ex vivo CT, RMS registration error in millimetres between histology and ex vivo CT and the total registration error between in vivo CT and histology calculated as square root of the sum of squares of the errors between individual steps. *CTin* computed tomography image obtained in vivo, *CTex* computed tomography image obtained ex vivo

### Ex vivo CT to histology co-registration

Table 3 shows the results of the ex vivo CT to histology registration error measurements. The mean ± SD accuracy was 0.86 ± 0.41 mm (mm). Figure 6 shows an example of the procedure for determining corresponding histology slices and ex vivo CT planes. RMS distances were calculated using either spine fiducial markers only (blue line) or the spine fiducial markers and an anatomical landmark (red line) that can be visualised in both CT and histology slices. Although there is approximate agreement between the two methods, the use of additional landmarks leads to more accurate identification, i.e. as indicated by the plane with the minimum RMS error, as the landmarks are often confined to a single (or two consecutive) axial slices. Although the measurements using the fiducial markers only (blue line) lead to smaller root mean square error (RMSE), both methods lead to a very similar global minimum position.

### Overall PET to histology error

**Fig. 8:**
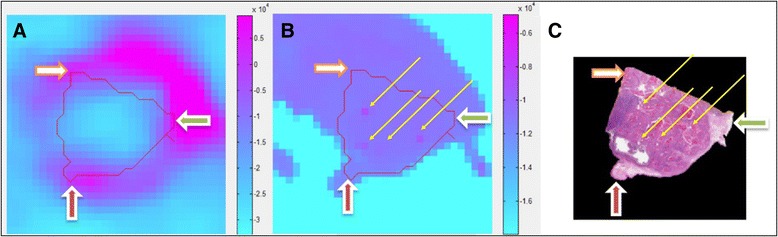
Registered PET, ex vivo CT and histology images. (**a**–**c**) show registered images of PET, ex vivo CT and histology from two different subjects. Regions in PET and ex vivo CT that correspond to histology are marked with a red outline. Yellow markers in (**c**) show the sea urchin spine markers that were clearly visible on the ex vivo CT and histology images

The average total registration error between PET and histopathology slices, assuming a PET-CT alignment accuracy of 1 mm, was 3.0 ± 0.7 mm. An example of the registration result between PET and histopathology slices is shown in Fig. 8.

## Discussion

We have developed and assessed a method for alignment of PET and histopathology slices obtained in routine pathology settings in six male larynxes with advanced HNSCC cancer. The average overall registration error between PET and histopathology was 3.0 ± 0.7 mm^2^ which is better than the 6.00-mm FWHM spatial resolution of the PET scanner.

One of the most challenging aspects of the process and one that future efforts should be focused on is the identification of the corresponding *z*-axis levels between the 2D histology and the 3D imaging data. When parallel consecutive slices are used, the slice thicknesses are accurately known and the identification of the *z*-axis can be performed by simple counting [27]. However, not all pathology departments have access to the equipment required, and the processing of these specimens would have to deviate from the local pathology laboratory routine. Contrary to previous attempts where the pathology routine had to be changed to introduce either whole-mount tissue sections or parallel consecutive slicing [27], the method outlined in this study affects the routine pathology workflow with the addition of one extra step, the insertion of the fiducial markers. This step, however, does not require extra personnel as it is not technically challenging and it can be performed easily by a member of the pathology team handling the specimen. In addition, it extends a procedure that has a total duration of many hours (from specimen removal after surgery to generation of tumour blocks for histopathology analysis) by few extra minutes only. For most routine pathology departments in which these kinds of study are performed, the pathologist performs parallel cutting with the use of a bandsaw, and the accuracy of this process depends on the experience of the pathologist and the tissue characteristics. With the use of optical images and the recorded anatomical information from the pathology records, we were able to identify the approximate level of each one of the tissue blocks on the ex vivo CT.

We employed sea urchin spines as fiducial markers which were clearly visible on both CT and histology images. The use of spines can add value for cases where bone tissue is absent.

‘Block-face’ images were obtained on both sides of the tissue blocks since tissues used for H&E staining were extracted from either side of the blocks and these images acted as a visual reference during registration. Even though block-face images are mainly used for shrinkage correction [4], the choice of rigid registration for this study obviated the exclusion of a histology-to-block-face step. However, as opposed to other organs, the lung for example [28], only small deformations occur inside the laryngeal cartilage area. An average shrinkage of 3 % before and after fixation has been calculated for the area included by the cartilage skeleton, which in most cases includes the tumour bulk [4]. This is because the larynx is an anatomical site with a strong and rigid skeleton, which helps to maintain the shape of the tissues inside it. In addition, it has been shown that the main shrinkage in the whole procedure occurs during the processing of the histological material to acquire the H&E sections from the thick slices and not from the fixation process [4]. Finally, excluding the use of block-face images avoided potential additional sources of error arising from (1) 3D volume reconstruction of block-face slices, (2) ex vivo CT to block-face registration and (3) histology to block-face registration.

Identification of reliable landmarks is challenging on PET, and therefore, the use of a PET-CT scanner was a major advantage. This is because an intermediate high-resolution ex vivo CT of the larynx specimen served as the reference data set that corresponded well with the in vivo CT data and also with the histopathology slice boundaries and/or edges. A disadvantage was the error due to PET-CT misalignment which was reported from literature and not measured, though the error introduced is likely to be small. A misalignment of ~1 mm may be expected in a worst case spatial calibration between PET and CT of the PET-CT scanner [29]. It was not possible to measure the PET-CT misalignment explicitly in this study; however, each PET-CT data set was visually verified by an expert clinician, and no further adjustment was deemed necessary in most of the data sets. Somer et al. [29] report an average PET-CT misalignment due to patient motion during the PET-CT scan as 5.4 mm; however, this figure was obtained primarily from measurement on the body and limbs and so is likely to greatly overestimate misalignments in the head and neck region. Nevertheless, further investigation of PET-CT misalignment would be useful, and the use of a rigid fixation device (e.g. a radiotherapy mask) may be advantageous.

Caldas-Magalhaes et al. [4] reported an average (in-plane) registration RMSE of 3.49 mm between PET and histopathology in laryngeal and hypopharyngeal cancer using a semi-automatic methodology, i.e. very similar to our overall error of 3.0 mm. Their histopathology samples were whole-mounted histology slices, obtained from a contiguously cut tissue block immersed into agarose medium whilst a meat-slicing machine was used for cutting. None of these methods are part of the routine pathology practice where usually only 30 × 21 mm slides are used and the specimen is sliced freehand without the provision of a supportive structure, such as the agarose medium. The carbon rods used as fiducial markers were not visible on histology, and therefore, manual landmarks were identified on the cartilage to drive the histology-to-block-face image registration. A study of the prostate by Park et al. [30] reported an average registration error of 7.7 mm between PET and histopathology using an in vivo MRI scan as the reference space. However, obtaining PET, CT and MR in vivo images may not be feasible in every clinical trial. Moreover, this study obtained contiguously cut 3-mm-thick parallel tissue slices with whole-mounted histology slices which again is not a routine pathology practice.

The current study suffers from a number of limitations. In particular, this is a specific case where tumour is surrounded by the larynx cartilage which prevents (in most cases) any unexpected deformation of the tumour during surgery, fixation and the slicing procedure.

## Conclusions

We have presented a registration methodology for accurate alignment between PET and histopathology slices that can be performed in routine pathology settings and that demonstrates an accuracy better than the spatial resolution of the PET scanner.
